# Effects of Propranolol, a β-noradrenergic Antagonist, on Memory Consolidation and Reconsolidation in Mice

**DOI:** 10.3389/fnbeh.2016.00049

**Published:** 2016-03-14

**Authors:** Hélène Villain, Aïcha Benkahoul, Anne Drougard, Marie Lafragette, Elodie Muzotte, Stéphane Pech, Eric Bui, Alain Brunet, Philippe Birmes, Pascal Roullet

**Affiliations:** ^1^Centre de Recherches sur la Cognition Animale, Centre de Biologie Intégrative, Université de Toulouse, Centre National de la Recherche Scientifique (CNRS), Université Paul Sabatier (UPS)Toulouse, France; ^2^Department of Psychiatry, Massachusetts General Hospital & Harvard Medical SchoolBoston, MA, USA; ^3^Department of Psychiatry, Douglas Mental Health University Institute and McGill UniversityMontréal, QC, Canada; ^4^Toulouse NeuroImaging Center, Université de Toulouse, Institut national de la santé et de la recherche médicale (INSERM), Université Paul Sabatier (UPS)Toulouse, France

**Keywords:** PTSD, consolidation, reconsolidation, propranolol, noradrenergic system, aversive memory, emotional valence

## Abstract

Memory reconsolidation impairment using the β-noradrenergic receptor blocker propranolol is a promising novel treatment avenue for patients suffering from pathogenic memories, such as post-traumatic stress disorder (PTSD). However, in order to better inform targeted treatment development, the effects of this compound on memory need to be better characterized via translational research. We examined the effects of systemic propranolol administration in mice undergoing a wide range of behavioral tests to determine more specifically which aspects of the memory consolidation and reconsolidation are impaired by propranolol. We found that propranolol (10 mg/kg) affected memory consolidation in non-aversive tasks (object recognition and object location) but not in moderately (Morris water maze (MWM) to highly (passive avoidance, conditioned taste aversion) aversive tasks. Further, propranolol impaired memory reconsolidation in the most and in the least aversive tasks, but not in the moderately aversive task, suggesting its amnesic effect was not related to task aversion. Moreover, in aquatic object recognition and location tasks in which animals were forced to behave (contrary to the classic versions of the tasks); propranolol did not impair memory reconsolidation. Taken together our results suggest that the memory impairment observed after propranolol administration may result from a modification of the emotional valence of the memory rather than a disruption of the contextual component of the memory trace. This is relevant to the use of propranolol to block memory reconsolidation in individuals with PTSD, as such a treatment would not erase the traumatic memory but only reduce the emotional valence associated with this event.

## Introduction

A newly acquired memory trace is initially labile and undergoes a consolidation process required to maintain it stable overtime (McGaugh, 1966, 2000). However, reactivation of a previously consolidated memory may render it labile again, introducing a need for restabilization called memory reconsolidation (Nader et al., 2000; Sara, 2000). Interfering with the reconsolidation processes impairs subsequent re-storage, inducing so-called amnesia in animals (for a systematic review, see Besnard et al., 2012). Combining targeted memory retrieval with a reconsolidation impairing agent may thus disrupt unwanted memories, and serve as treatment for individuals suffering from a pathogenic memory, such as in post-traumatic stress disorder (PTSD), which involve impairment of memory processes in their pathophysiology (Brewin, 2011).

The noradrenergic system is critical in modulating memory processes, and stimulation of β-noradrenergic receptors has been found to facilitate emotional and non-emotional memory consolidation (McGaugh, 2004; Roozendaal et al., 2008) as well as reconsolidation (Dębiec et al., 2011). Noradrenergic enhancement, and specifically enhancement of the β-noradrenergic signaling during memory reconsolidation has been suggested to increase the strength of emotional memory, and has thus been suggested to contribute to the persistence of traumatic memories (Dębiec et al., 2011). Moreover, a hyper-noradrenergic state has been implicated in the pathophysiology of PTSD (Strawn and Geracioti, 2008). In this context, the β-noradrenergic receptors antagonist propranolol has generated considerable interest as an agent for dampening emotional and traumatic memories in healthy humans and in individuals with PTSD.

In healthy humans, propranolol has been found to impair the consolidation of emotional items memory (Cahill et al., 1994; van Stegeren et al., 2005), and of fear memory in a contextual fear conditioning test (Grillon et al., 2004). Administered concurrently to fear conditioned memory reactivation, propranolol has been shown to reduce subsequent fear related to the cue, leaving the declarative memory associated to the cue intact (Kindt et al., 2009). Despite a few negative studies (e.g., Bos et al., 2014), the majority of them reported successful propranolol-induced disruption of reconsolidation, that persisted at least 1 month and resisted to fear reinstatement (Soeter and Kindt, 2010, 2012; for a meta-analytic review, see Lonergan et al., 2013).

From a therapeutic perspective, propranolol has been tested for phobia (Soeter and Kindt, 2015), substance abuse (Lonergan et al., 2015) and more interestingly for PTSD. Initially, propranolol was used to prevent the consolidation of the traumatic memories in acutely traumatized individuals. Two studies reported a reduction of PTSD rate and symptoms in patients (Pitman et al., 2002; Vaiva et al., 2003). However, targeting the consolidation processes proved to be impractical because of the short time-window of action for blocking consolidation in the aftermath of trauma, and because not all trauma-exposed individuals will develop PTSD. A way to get around these issues is to act during memory reactivation when the memory trace is labile again. Thus, using a randomized controlled design, Brunet et al. (2008) administered a single day of propranolol following traumatic memory reactivation and found that this treatment reduced, 1 week later, physiologic responses to mental imagery of the traumatic event in PTSD patients. However, a recent study failed to replicate this beneficial effect with a single reactivation session associated to propranolol (Wood et al., 2015). In a series of three open label trials, a PTSD remission rate of up to 71% was observed after six sessions of trauma recollection under the influence of propranolol (Brunet et al., 2011), with significant reduction in physiological responding including at the 6-month follow-up (Brunet et al., 2014).

While blockade of memory reconsolidation thus appears as a promising treatment approach to further test and explore, many important questions remain unanswered. For example, the nature and extent of the memory impairment remain to be clarified so as to effectively and specifically target memories involved in the pathophysiology of disorders, without affecting others. In particular, the types of memory (e.g., traumatic, contextual) on which propranolol exerts its effects are still unknown. Translational research requires, in parallel to the human clinical research, basic research to be conducted in animals to clarify the effects of reconsolidation impairment with propranolol.

The literature remains unclear and often contradictory regarding the amnesic effects of propranolol in animal models. Some research reported that propranolol impaired consolidation in tasks as varied as the passive avoidance (Gallagher et al., 1977), the Morris water maze (MWM; Cahill et al., 2000) or the object recognition (Conversi et al., 2014), while others using the same tasks failed to do so (Lalumiere et al., 2004; Dornelles et al., 2007; Row and Dohanich, 2008; Okamura et al., 2011; Palotai et al., 2014). Data regarding the effects of propranolol on memory reconsolidation have been more consistently reported than its effect on consolidation. Administration of propranolol at the time of retrieval has thus been found to dampen contextual and auditory fear memory (Dębiec and LeDoux, 2004, 2006; Muravieva and Alberini, 2010), spatial memory in a radial maze test (Przybyslawski et al., 1999), and appetitive memories (Bernardi et al., 2006; Diergaarde et al., 2006; Robinson and Franklin, 2007). However, some studies failed to report an amnesic effect associated with propranolol administration in passive avoidance (Muravieva and Alberini, 2010), contextual conditioning (Gazarini et al., 2013), and object recognition (Maroun and Akirav, 2008). These conflicting results might be due to differences in methods and procedures between species or strain of animals, and in doses of drug or routes of administration (systemic vs. central into a brain region).

Thus, to date, the effects of propranolol on human and animal memories remain to be clarified. It is becoming increasingly important to understand the effect of propranolol on various types of memories in order to successfully guide treatment development for mental disorders including PTSD.

In the present study, we therefore sought to determine which aspects of memory were affected by propranolol by using a broad range of behavioral tests performed in the same lab with the same strain of mice so as to compare directly and unambiguously in the same conditions all behavioral results. These tasks differ in their spatial, associative, of aversive characteristics ranging from very aversive (passive avoidance, conditioned taste aversion), moderate (MWM) to non aversive task (object recognition and object location). We found that propranolol-induced amnesia was not related to the degree of task aversiveness or the spatial or associative nature of the tasks. To further clarify our results, we then developed a series of new behavioral experiments, in order to examine independently memory problem (i.e., contextual amnesia) and emotional state (i.e., memory valence, detachment). In these new procedures, in which animals were forced to behave, propranolol had no effect on contextual memory.

Finally, we also sought to compare the propranolol-induced amnesia in consolidation vs. reconsolidation within the same experimental conditions. As propranolol acts by blocking the activation of β-noradrenergic receptors by noradrenaline, in these studies, it will be possible to know if endogenous noradrenaline and specifically β-noradrenergic signaling play the same critical role in memory consolidation and reconsolidation, and if this implication is the same whatever be the tasks used. In different studies, propranolol was found to induce amnesia only if injected during the memory reconsolidation, but not during initial memory consolidation in very aversive learning as the passive avoidance task (Przybyslawski et al., 1999) or the auditory fear conditioning task (Dębiec and LeDoux, 2004). We complemented these experiments with other aversive tasks as well as with non-aversive learning task, and found this differential effect of propranolol on reconsolidation and consolidation, only in highly aversive task.

## Materials and Methods

### Animals

A total of 368 CD1 male mice (IFFA CREDO, Lyon, France) were housed in groups of five in standard breeding cages placed in a rearing room at a constant temperature under diurnal conditions (light-dark: 08:00-20:00), with food and water *ad libitum*. Every possible effort was made to minimize animal suffering and all experiments were performed in strict accordance with the recommendations of the European Union (86/609/EEC) and the French National Committee (87/848). All animal procedures were approved by the University Animal Care Committee of Toulouse (FRBT C2EA-01).

### Behavioral Testing

The number of mice per group ranged between 8 and 14. Three- to four-months old mice were familiarized with the experimenter and all experiments were performed during the light phase. All the behavioral groups were independent, i.e., each mouse was tested in only one experiment.

An informed choice of behavioral procedures is essential and we chose only paradigms well suited to study both the initial consolidation and the subsequent reconsolidation phase given their rapid acquisition. For the reconsolidation studies, we chose well established behavioral procedures and reactivation protocols previously used in different memory reconsolidation studies. Moreover, an essential control in reconsolidation studies involves the administration of the drug treatment without the reactivation session. Thus, each time we found a propranolol effect, we added a control experiment to determine if the propranolol-induced reconsolidation impairment was specific to an actively retrieved memory. To achieve that, we used exactly the same protocol as in the reconsolidation experiments except that mice did not undergo reactivation and that injections were performed in the animal room.

Mice were subjected to the following behavioral tests.

#### Passive Avoidance Task

The apparatus consisted of a rectangular box divided into an illuminated “safe” compartment and a dark “shock” compartment. Subjects were individually placed into the illuminated compartment and when they entered completely inside the dark compartment, a foot shock (280 μA for 2 s) was administered. Subsequently, the mouse was placed in its home cage. For the consolidation procedure, retention was measured 24 h later by placing the mouse in the light compartment and measuring the latency to enter the dark compartment and the percentage of avoidance of the dark side. For the reconsolidation procedure, to reactivate memory, 24 h after acquisition, we placed the mouse during 60 s into the illuminated compartment (open door). The entry into the dark compartment during reactivation was chosen as an exclusion criterion and for this reason three mice were removed from the experiments. The probe test (PT) was realized 24 h later (Jobim et al., 2012).

#### Conditioned Taste Aversion Test (CTA)

The mice were initially deprived of water for 24 h and then habituated in a new cage to drink water from a graduated bottle for 30 min/day for 6 days (days 1–6). After a stable water consumption baseline was reached, the animals were randomly divided into treatment groups and acquisition of CTA was performed. For conditioning (day 7), the water bottle was replaced by a bottle containing a 1% saccharin solution and the mice were allowed to drink for 30 min. Thirty minutes after completion of saccharin intake, the animals were injected intraperitoneally with lithium chloride (LiCl; 125 mg/kg) that induces nausea and produces robust CTA (Miranda et al., 2003; Tuerke et al., 2012). During the first hour following the injection, the behavior of the mice in their home cage was observed to control whether nausea had occurred, and then a normal behavior was recovered within 1 h. In our experimental conditions, the gastric malaise was observed in all mice treated with NaCl or propranolol and this malaise was only visible during 10–20 min after the injection. As the duration of malaise was short, propranolol was always active during the consolidation of the CTA. In the choice test 1 day later (day 8), mice were placed back into the same cage and had access to bottles containing either water or saccharin for a period of 30 min. The volume drunk in each bottle was recorded.

For the reconsolidation procedure, the habituation and acquisition phases were identical to the consolidation procedure (days 1–7). For the next 3 days after acquisition (days 8–10), mice received water as during the habituation phase. Twenty-four hours later (day 11), to reactivate memory, we placed the mouse for 1 min into the cage with the bottle of saccharin (Bahar et al., 2004). The choice test took place 24 h after reactivation (day 12).

#### Morris Water Maze Test (MWM)

Spatial memory testing was conducted as described (Florian and Roullet, 2004). Briefly, mice were introduced to a circular pool (110 cm in diameter) filled with water made opaque. Subjects were trained to locate the hidden platform, which was submerged 0.5 cm below the water. One mass-training procedure was performed. The procedure included one training session composed of four blocks, each consisting of three consecutive trials. The phase between consecutive blocks was 15–20 min long, during which the mouse was returned to its home cage. The total duration of this training phase do not exceed 1 h 20 min. For the consolidation procedure, 24 h post acquisition, memory was assessed during a single 1 min PT in the absence of the platform. The number of annulus crossings, defined as the number of times a mouse crossed an ideal circle (14 cm diameter) located at the original platform location and the three equivalent areas in each of the other quadrants were analyzed. The number of annulus crossings could reveal the strategy used whilst searching for the platform, while the number of target annulus crossings would determine if mice had learned the target’s location.

For the reconsolidation procedure, 24 h after training, a reactivation trial (R) consisting in an additional learning trial, was performed (De Jaeger et al., 2014). The PT took place 24 h after reactivation.

In the MWM, the level of stress is dependent on the water temperature. In the first experiment, the temperature of the water is relatively high (23 ± 1°C) and in this condition, MWM might be considered as a mildly stressful situation. In the second experiment, the temperature of the water is low (19 ± 1°C) and MWM might be considered as a highly stressful situation (Sandi et al., 1997).

#### Object Recognition Task

The procedure consisted of three different phases as described previously (Goodman et al., 2010). Briefly, a familiarization phase in which each mouse was placed in the empty square open-field for 10 min. A sample phase, 24 h later, in which two identical objects were placed in the middle of the open-field. The test phase, for the consolidation procedure, 24 h later whereby mice were reintroduced into the arena and exposed to two objects, a familiar object and a novel object, to test recognition memory. The percent time spent exploring the novel object was calculated as a preference index to measure novel object recognition. For the reconsolidation procedure, 24 h after training, a 2 min reactivation phase, identical to the sample phase, was performed (Rossato et al., 2007).

#### Object Location Task

The same open-field with the same environment was used as that of the object recognition task. Similar procedures were employed except that for the test phase, one of the two identical objects was moved to a novel location (Goodman et al., 2010). The percent time spent exploring the displaced object was calculated as a preference index to measure spatial memory.

Finally, two novel behavioral tests were specifically designed for this study, the aquatic object recognition and object location tasks.

#### Aquatic Object Recognition Task

Mice were introduced into the circular pool (23°C) previously described for the MWM, except that the distal cues were removed. An object was suspended 13 cm above the 0.5 cm-submerged platform. In this new behavioral task, the mouse had to learn the association between the object and the presence of the platform. One mass-training procedure composed of four blocks of three consecutive trials was performed. The total duration of this training phase do not exceed 1 h 20 min. For each block, platform and object were placed in a different quadrant of the pool. Twenty-four hours after training, a reactivation trial (R) consisting in an additional learning trial, was performed. The PT took place 24 h after reactivation. The platform was removed and the familiar object was placed in the northern quadrant and a new object was placed in the opposite quadrant. The mouse had to dissociate the two objects as in the classic version of the object recognition task and had to choose the location associated with the familiar object. The number of annulus (a 14 cm diameter circle located below the objects) crossings was analyzed and the percent number of crossing below the familiar object was calculated as a preference index to measure familiar object recognition.

#### Aquatic Object Location Task

The same pool (23°C) was used as that of the aquatic object recognition task but this time some distal cues were added in the environment. In this task, the mouse must also learn the association between the object and the presence of the platform. But in this new procedure, the platform is always in the same place during the four blocks of three trials. Twenty-four hours after training, a reactivation trial (R) consisting in an additional learning trial, was performed. The PT took place 24 h after reactivation. During this test, the platform was removed and a second object, identical to the first one, was placed in the opposite quadrant. The mouse had to differentiate the two spatial locations as in the classic version of the object location but this time, had to choose the old location to find the platform. The number of annulus crossings was analyzed and the percent number of crossing below the non-displaced object was calculated as a preference index to measure familiar object location.

### Drug and Injections

DL-Propranolol, an antagonist of β-noradrenergic receptors that crosses the blood-brain-barrier, obtained from Sigma-Aldrich (France) was prepared in 0.9% saline (NaCl) and injected intraperitoneally at a dose of 10 mg/kg (Przybyslawski et al., 1999; Conversi et al., 2014). NaCl and propranolol were both administered at a volume of 10 mL/kg. Mice received injections of propranolol or NaCl immediately after training for the consolidation procedure except for the CTA test in which the drugs were injected 25 min after the presentation of saccharin and therefore 5 min before LiCl injection. For the reconsolidation procedure, mice received injections of propranolol or NaCl just after reactivation, or the day following learning for the no-reactivation procedure. The memory test took place 24 h after injection. Consequently, animals were never tested under the influence of propranolol but the day after injection, when the drug was no longer present in the organism.

### Statistical Analysis

SYSTAT 9.0 statistical software package was used for data analysis. The results were expressed as mean ± SEM and analyzed using one or two-way analyses of variance (ANOVAs), or repeated measures ANOVAs when appropriate. Partial eta squared ($\eta_{p}^{2}$) was reported for each significant comparison as a measure of effect size for significant results in ANOVAs. The alpha level of significance was set at 0.05 (two-sided tests). *Post hoc* multiple comparison tests were carried out when allowed, using Tukey’s Honestly Significant Distance (HSD) test. For the passive avoidance task, the percentage of avoidance of the dark compartment was analyzed between groups using *χ^2^* tests.

## Results

### Passive Avoidance

The baseline latency to enter the dark compartment during acquisition (i.e., before treatment), did not differ between the two groups of mice for the consolidation [NaCl: 17.3 s ± 3.1; Propranolol: 15.4 s ± 1.9; *F*_(1,26)_ = 0.262; *p* = 0.613], reconsolidation [NaCl: 19.2 s ± 4.5; Propranolol: 18.5 s ± 6.4; *F*_(1,20)_ = 0.008, *p* = 0.928] and no-reactivation [NaCl: 15.5 s ± 4.0; Propranolol: 19.5 s ± 12.018; *F*_(1,15)_ = −1.312; *p* = 0.209] procedures.

**Figure 1:**
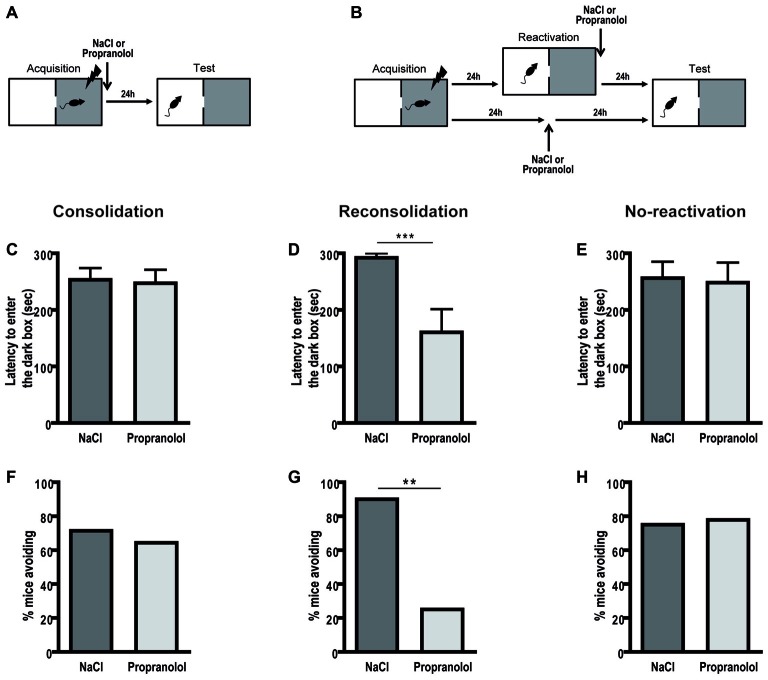
**Propranolol disrupts memory reconsolidation in the passive avoidance task.** Schematic of the behavioral protocols for the consolidation **(A)** and reconsolidation and no-reactivation **(B)** procedures. In the consolidation procedure (*n* = 14 per group), there was no memory deficit in propranolol-injected mice for the latency (±SEM) to enter the dark box **(C)**, nor for the percentage of avoidance of this aversive box **(F)**. However, for the reconsolidation procedure, propranolol-injected mice (*n* = 8) exhibited decreased memory performance relative to control mice (*n* = 10) **(D,G)**. In the no-reactivation procedure, propranolol-injected mice (*n* = 9) showed the same level of avoidance as control mice (*n* = 8) **(E,H)**. **p < 0.01; ***p < 0.001 NaCl vs. Propranolol.

During the test, in the memory consolidation procedure (Figure 1A), there was no effect of treatment on the latency to enter the dark side of the box [*F*_(1,26)_ = 0.038; *p* = 0.846] and on the percentage of avoidance of this dark side [*χ^2^* = 0.158; *p* = 0.691] as illustrated in Figures 1C,F. However, propranolol injected after reactivation in the passive avoidance task (Figure 1B) caused a marked performance decrement during the test session (Figures 1D,G). Propranolol-injected mice exhibited shorter latencies to enter the dark compartment during the retention test [$\eta_{p}^{2}$ = 0.433; *F*_(1,16)_ = 12.203; *p* = 0.003] and avoided less this aversive compartment than controls [*χ^2^*= 7.462; *p* = 0.006]. Moreover, propranolol administered without any reactivation (Figures 1B,E,H) did not affect memory performance [latencies: *F*_(1,15)_ = 0.029; *p* = 0.867, avoidance: *χ^2^* = 0.017; *p* = 0.896] demonstrating an amnesic effect specific to memory reconsolidation in this aversive task.

### Conditioned Taste Aversion

During acquisition and reactivation, we did not find any difference in liquid consumption between the two groups of mice, for the consolidation [acquisition: *F*_(1,12)_ = 1.687; *p* = 0.218], reconsolidation [acquisition: *F*_(1,14)_ = 0.578; *p* = 0.459; reactivation: *F*_(1,14)_ = 1.647; *p* = 0.220] and no-reactivation [acquisition: *F*_(1,14)_ = −0.260; *p* = 0.799] procedures. Thus before treatment, the two groups of mice similarly learned this task.

**Figure 2:**
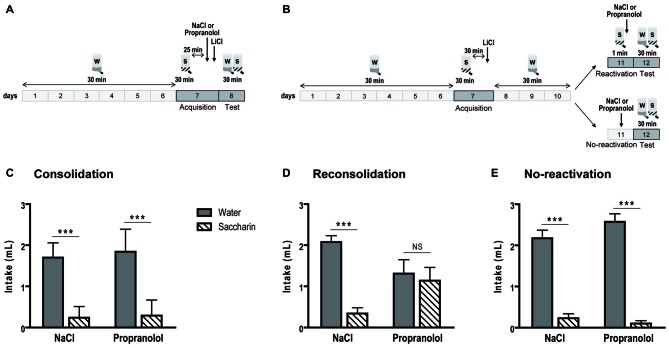
**Propranolol disrupts memory reconsolidation in the conditioned taste aversion task.** Schematic of the behavioral protocols for the consolidation **(A)** and reconsolidation and no-reactivation **(B)** procedures. Mean consumption (±SEM) of water (gray) and saccharin (white hachured) in the choice test is represented. **(C)** In the consolidation procedure (*n* = 7 per group), a clear preference for the water was displayed for control but also for propranolol-injected mice. **(D)** On the contrary, for the reconsolidation procedure (*n* = 8 per group), control mice avoided saccharin while propranolol-injected mice showed no preference for water or saccharin. **(E)** In the no-reactivation procedure, there was not any memory deficit in propranolol-injected mice thus a preference for the water was displayed in both group of mice (*n* = 8 per group). ***p < 0.001 Water vs. Saccharin.

In the consolidation procedure (Figures 2A,C), during the choice test, an ANOVA revealed no treatment effect [*F*_(1,24)_ = 0.366; *p* = 0.551], no interaction between treatment and liquid [*F*_(1,24)_ = 0.113; *p* = 0.739], but an important preference for water over saccharin [$\eta_{p}^{2}$ = 0.796; *F*_(1,24)_ = 93.843; *p* < 0.001]. In fact, mice consumed more water than saccharin among control (*p* < 0.001), as well as among propranolol-treated mice (*p* < 0.001).

In the reconsolidation procedure (Figures 2B,D), we also obtained no main effect for treatment [*F*_(1,28)_ = 0.005; *p* = 0.943] and a global preference for water over saccharin [$\eta_{p}^{2}$ = 0.327; *F*_(1,28)_ = 13.618; *p* < 0.001]. However, in this procedure, there was an important treatment × liquid interaction [$\eta_{p}^{2}$ = 0.245; *F*_(1,28)_ = 9.090; *p* = 0.005]. While there was a clear water preference in control mice (*p* < 0.001), propranolol-injected mice consumed similar quantities of water and saccharin (*p* = 0.963). When memory was not reactivated before propranolol injection (Figures 2B,E), mice strongly preferred water [$\eta_{p}^{2}$ = 0.894; *F*_(1,24)_ = 203.440; *p* < 0.001]. We did not observe any treatment effect [*F*_(1,24)_ = 0.769; *p* = 0.389] and interaction between treatment and liquid [*F*_(1,24)_ = 2.917; *p* = 0.101].

Once again, in these experiments, we found a propranolol amnesic effect only on memory reconsolidation.

### Morris Water Maze at 23°C

**Figure 3:**
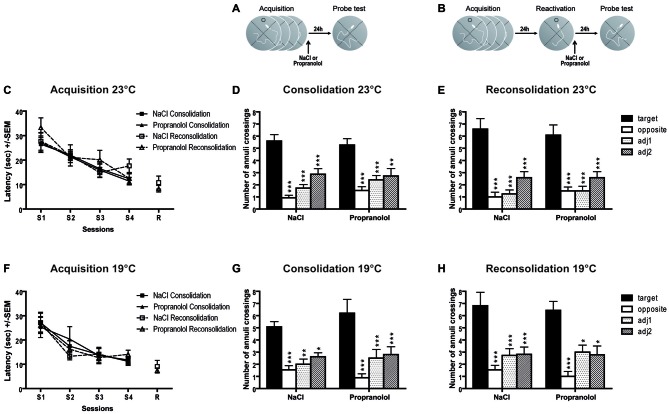
**Propranolol has no effect on memory reconsolidation in the Morris water maze (MWM) task.** Schematic of the behavioral protocols for the consolidation **(A)** and reconsolidation **(B)** procedures. 23°C procedure **(C–E).** Throughout training sessions **(C)**, mice learned equally well to locate the hidden platform and exhibited decreasing latencies (±SEM) over blocks of trials in the two different behavioral procedures (consolidation and reconsolidation). The number of annuli crossings (±SEM) during probe test (PT) in the consolidation (*n* = 15 per group; **D**) and reconsolidation (*n* = 12 per group; **E**) procedures are represented. 19°C procedure **(F–H)**. For this more aversive procedure with cold water, we found exactly the same results as in the 23°C procedure i.e., mice learned equally well to locate the hidden platform during training sessions **(F)**, and all groups of mice showed similar preference for the target zone in the consolidation (NaCl: *n* = 10; Propranolol: *n* = 14; **G**) and in the reconsolidation (NaCl: *n* = 9; Propranolol: *n* = 11; **H**) procedures. target vs. others, *p < 0.05; **p < 0.01; ***p < 0.001.

Concerning the latency to find the hidden platform during acquisition (Figure 3C), repeated measures ANOVA revealed a significant session effect [$\eta_{p}^{2}$ = 0.367; *F*_(3,150)_ = 21.688; *p* < 0.001] but no pre-treatment effect [*F*_(1,50)_ = 0.072; *p* = 0.789], nor procedure effect [*F*_(1,50)_ = 1.515; *p* = 0.224]. These data confirmed that, in general, before treatment all groups of mice similarly learned the position of the platform and displayed the same level of performance during the four learning sessions.

In the consolidation procedure (Figures 3A,D), propranolol injection immediately after acquisition did not impair retention during the PT 24 h later. In this experiment, a two-way ANOVA revealed a significant quadrant effect [$\eta_{p}^{2}$ = 0.487; *F*_(3,112)_ = 35.489; *p* < 0.001] but no treatment effect (*F*_(1,112)_ = 0.430; *p* = 0.513), and no treatment × quadrant interaction [*F*_(3,112)_ = 0.693; *p* = 0.558].

In the reconsolidation procedure (Figures 3B,E), an ANOVA conducted on the latency to reach the platform during reactivation did not find any pre-treatment effect [*F*_(1,22)_ = 0.906; *p* = 0.351]. During the PT, propranolol injections immediately after reactivation did not impair retention during the PT 24 h later. A two-way ANOVA revealed no significant effect between NaCl- and propranolol-injected mice [*F*_(1,88)_ = 0.025; *p* = 0.874] on the total number of annulus crossings, but a significant quadrant effect [$\eta_{p}^{2}$ = 0.556; *F*_(3,88)_ = 36.807; *p* < 0.001] and no significant treatment × quadrant interaction [*F*_(3,88)_ = 0.297; *p* = 0.827], indicating that the profile of exploration of the different quadrants was the same in the two groups of mice.

Because we used a relatively high water temperature (23°C) and the non-effect of propranolol might be linked to this low magnitude stressful condition, we chose to replicate the MWM with water at 19°C, a more aversive procedure.

### Morris Water Maze at 19°C

Concerning the latency to find the hidden platform during acquisition (Figure 3F), repeated measures ANOVA revealed a significant session effect [$\eta_{p}^{2}$ = 0.348; *F*_(3,120)_ = 21.293; *p* < 0.001], but no pre-treatment effect [*F*_(1,40)_ = 0.049; *p* = 0.825], nor procedure effect [*F*_(1,40)_ = 0.266; *p* = 0.609], suggesting a similar and good level of performance in the two groups.

During the PT for the consolidation procedure (Figures 3A,G), propranolol injections did not impair memory retention during the PT 24 h later. A two-way ANOVA revealed a significant quadrant effect [$\eta_{p}^{2}$ = 0.444; *F*_(3,84)_ = 22.448; *p* < 0.001] but no treatment effect [_1,84_ = 0.631; *p* = 0.429], and no treatment × quadrant interaction [*F*_(3,84)_ = 0.834; *p* = 0.479].

In the reconsolidation procedure (Figures 3B,H), an ANOVA conducted on the latency to reach the platform during the reactivation trial did not reveal any treatment effect [*F*_(1,18)_ = 0.312; *p* = 0.585]. A two-way ANOVA revealed no significant effect between NaCl- and propranolol-injected mice [*F*_(1,72)_ = 0.126; *p* = 0.724] on the total number of annulus crossings, but a significant quadrant effect [$\eta_{p}^{2}$ = 0.488; *F*_(3,72)_ = 22.221; *p* < 0.001] and no significant treatment × quadrant interaction [*F*_(3,72)_ = 0.141; *p* = 0.935] indicating that the profile of exploration of the quadrants was the same in the two groups of mice. With this new more aversive MWM procedure, we obtained exactly the same results than with the less aversive MWM procedure, i.e., no effect of propranolol injection on the consolidation and reconsolidation processes.

Taken together, our results suggest that propranolol injections have no effect on spatial memory consolidation and reconsolidation. In this experiment and in many others studies (Przybyslawski et al., 1999; Dębiec and LeDoux, 2004; Muravieva and Alberini, 2010), propranolol injection blocked the reconsolidation memories of an aversive nature. It is possible that the lack of effect was due to the MWM being less aversive than passive avoidance or CTA. In other words, it is possible that propranolol only exerts an effect on aversive memory reconsolidation. In order to test this hypothesis, we used two different non-aversive paradigms, the object recognition task, a non-spatial and non-associative task, and the object location task, a spatial but non-associative task.

### Object Recognition

During the training phase of the object recognition test, and thus before treatment, we verified that there was no difference in the total amount of time spent in exploration of the two identical objects between propranolol-treated and saline control mice for consolidation [NaCl: 160.5 s ± 16.8; Propranolol: 172.6 s ± 10.3; *F*_(1,34)_ = 0.030; *p* = 0.863], reconsolidation [NaCl: 118.38 s ± 12.01; Propranolol: 114.44 s ± 11.42; *F*_(1,36)_ = 0.082; *p* = 0.776], and no-reactivation [NaCl: 107.1 s ± 15.3; Propranolol: 112.7 s ± 19.8; *F*_(1,28)_ = 0.090; *p* = 0.766] procedures.

**Figure 4:**
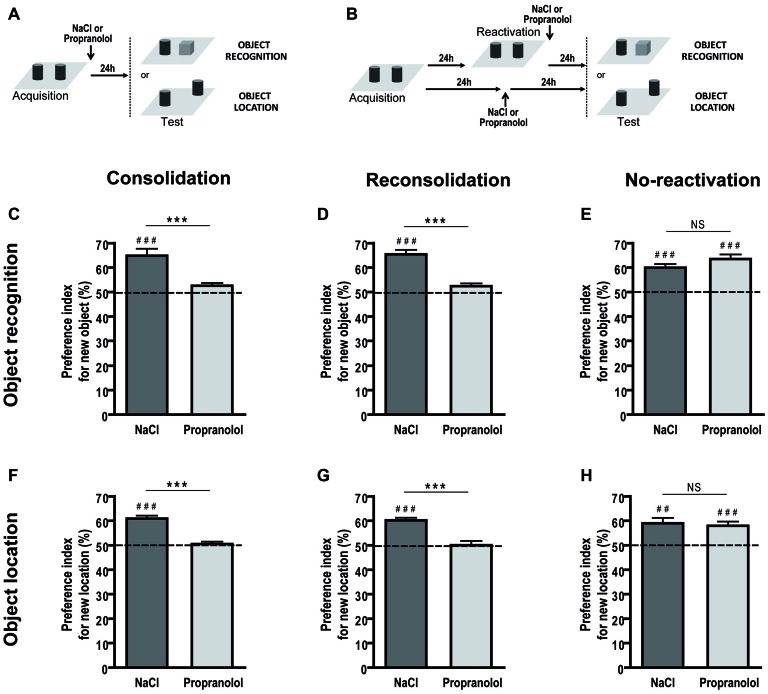
**Propranolol impairs the memory reconsolidation in the object recognition and object location tasks.** Schematics of the behavioral protocols of the object recognition and object location tasks for the consolidation **(A)** and reconsolidation and no-reactivation **(B)** procedures. Performances are expressed as the group mean (±SEM) preference index. The horizontal line represents equal exploration of the two objects. For the consolidation (*n* = 9 per group; **C**) and reconsolidation (NaCl: *n* = 10; Propranolol: *n* = 9; **D**) procedures of the object recognition task, control mice spent significantly more time exploring the new object than the familiar one. For the consolidation (NaCl: *n* = 13; Propranolol: *n* = 14; **F**) and reconsolidation (NaCl: *n* = 14; Propranolol: *n* = 13; **G**) procedures of the object location task, NaCl-injected mice spent significantly more time exploring the displaced object than the non displaced one. Mice with propranolol injection during consolidation or reconsolidation presented severe deficits in these two spatial tasks and were not able to distinguish the new or displaced object. For the no-reactivation procedures, NaCl- but also propranolol-injected mice showed similar preference for the familiar or non-displaced object as compared to the new or displaced object in the object recognition (NaCl: *n* = 7; Propranolol: *n* = 8; **E**) and object location (NaCl: *n* = 8; Propranolol: *n* = 9; **H**) tasks respectively. ^##^*p* < 0.01; ^###^*p* < 0.001 index vs. chance level; 50%. ***p < 0.001 NaCl vs. Propranolol; NS, non significant.

Concerning the consolidation (Figure 4A), during the retention test, as expected, control mice spent significantly more time exploring the novel object as opposed to the familiar one, so that the preference index was significantly different from chance level (50%; 64.811 ± 2.90%; *p* = 0.001). In contrast, the preference index for propranolol-injected mice was not different from chance level (52.505 ± 1.17%; *p* = 0.086; Figure 4C). An ANOVA conducted on the preference index revealed a treatment effect [$\eta_{p}^{2}$ = 0.491; *F*_(1,16)_ = 15.406; *p* < 0.001].

In the reconsolidation procedure (Figure 4B), mice injected with NaCl explored longer the new object than the familiar one (65.450 ± 1.753%; *p* < 0.001) contrary to the propranolol-injected mice (52.392 ± 1.14%; *p* = 0.096), suggesting that the experimental animals did not recognize the object [$\eta_{p}^{2}$ = 0.686; *F*_(1,17)_ = 37.081; *p* < 0.001; Figure 4D]. When no memory reactivation was performed prior to treatment (Figure 4E), propranolol did not influence exploration time [*F*_(1,13)_ = 2.099; *p* = 0.171]. Both NaCl (59.934 ± 3.963%; *p* < 0.001) and propranolol-treated (63.470 ± 5.276%; *p* < 0.001) mice explored longer the new object than the familiar object.

These results suggest that propranolol-injected mice may have exhibited a non-spatial specific memory deficit in this non-aversive and non-associative task.

### Object Location

The total time spent during task acquisition exploring the objects did not differ between propranolol-injected and saline control mice in the consolidation [NaCl: 159.1 s ± 8.5; Propranolol: 158.1 s± 9.4; *F*_(1,52)_ = 0.009; *p* = 0.927], reconsolidation [NaCl: 108.3 s ± 7.2; Propranolol: 105.4 s ± 6.8; *F*_(1,52)_ = 0.035; *p* = 0.852] and no-reactivation procedures of object location [NaCl: 138.4 s ± 26.7; Propranolol: 146.4 s ± 27.4; *F*_(1,32)_ = 0.087; *p* = 0.770].

Regarding the consolidation procedure (Figure 4A), control mice spent significantly more time exploring the object that had been displaced to a new location than the object that had remained in a familiar location. Thus, control mice exhibited an exploratory preference index that was clearly different from chance level (50%; 60.935 ± 1.14%; *p* < 0.001). In contrast, propranolol-treated mice exhibited an exploratory preference index that was not significantly different from chance (50.499 ± 0.951%; *p* = 0.609; Figure 4F). ANOVA conducted on the preference index revealed a significant treatment effect [$\eta_{p}^{2}$ = 0.665; *F*_(1,25)_ = 49.622; *p* < 0.001].

For the reconsolidation procedure (Figure 4B), mice injected with NaCl explored longer the displaced object than the non-displaced one (60.156 ± 1.23%; *p* < 0.001) contrary to the propranolol-injected mice (50.098 ± 1.70%; *p* = 0.609) indicating that these experimental animals were not able to recognize the displaced object [$\eta_{p}^{2}$ = 0.484; *F*_(1,25)_ = 23.485; *p* < 0.001; Figure 4G]. This deficit was specific to reactivated memory as in the non-reactivation procedure (Figure 4H), both groups of mice recognized the displaced object (NaCl: 59.030 ± 6.257%; *p* = 0.005; Propranolol: 59.980 ± 5.059%; *p* = 0.001), without any influence of the treatment [*F*_(1,15)_ = 0.146; *p* = 0.708].

In this non-aversive and non-associative task, propranolol-injected mice exhibited a spatial memory deficit compared to controls.

### Aquatic Object Recognition

**Figure 5:**
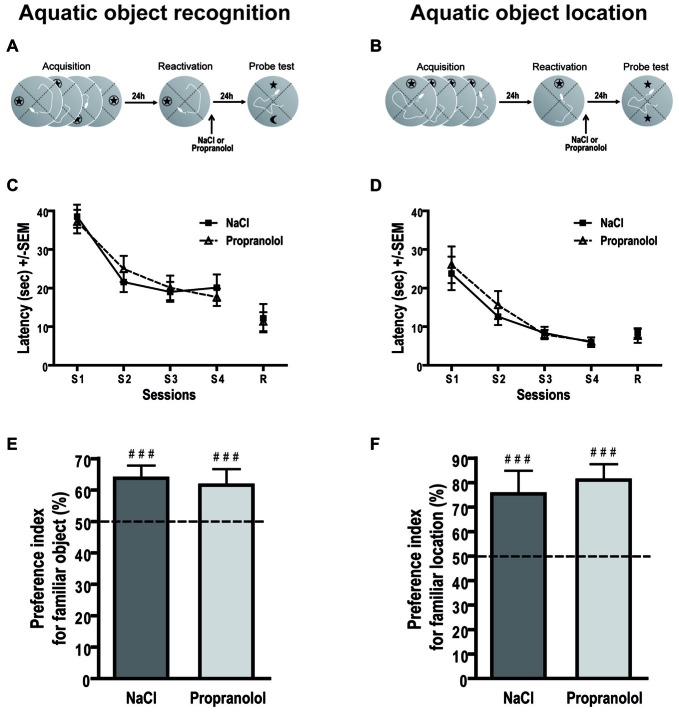
**Propranolol does not impair memory reconsolidation in the aquatic object recognition and object location tasks.** Schematics of the behavioral protocols of the aquatic object recognition **(A)** and object location **(B)** tasks. Throughout training sessions, mice learned equally well to find the hidden platform and exhibited decreasing latencies (±SEM) over blocks of trials in the aquatic object recognition **(C)** and location **(D)** tasks. Performances during PT are expressed as the group mean (±SEM) preference index. The horizontal line represents equal exploration of the two objects. **(E)** For reactivation procedure of the aquatic version of the object recognition task, NaCl- but also propranolol-injected mice showed similar preference for the familiar object as compared to the new object (*n* = 11 per group). **(F)** For reactivation procedure of the aquatic version of the object location task, we obtained the same behavioral profile i.e., the two groups of mice spent significantly more time below the object that had remained in a familiar location than the object that had been introduced to a new location (*n* = 8 per group). ^###^*p* < 0.001 index vs. chance level; 50%.

Concerning the latency to find the hidden platform during acquisition for the aquatic object recognition test (Figures 5A,C), repeated measures ANOVA revealed a significant session effect [$\eta_{p}^{2}$ = 0.513; *F*_(3,60)_ = 21.105; *p* < 0.001] but no pre-treatment effect [*F*_(1,20)_ = 0.160; *p* = 0.694], nor session pre-treatment interaction [*F*_(3,60)_ = 1.545; *p* = 0.212]. An ANOVA conducted on the latency to reach the platform during the reactivation trial also revealed no treatment effect [*F*_(1,20)_ = 0.008; *p* = 0.930]. Thus, before treatment all groups of mice displayed the same level of performance during the four learning sessions.

During PT (Figure 5E), all mice crossed significantly more time below the familiar object than below the new object. ANOVA conducted on the preference index revealed no treatment effect [*F*_(1,20)_ = 0.111; *p* = 0.743]. Control and propranolol mice exhibited an exploratory preference index that was clearly different from chance level (50%; NaCl: 63.714 ± 4.04%; *p* < 0.001; Propranolol: 61.541 ± 5.13%; *p* < 0.001). Thus, in this new behavioral task, propranolol mice presented no memory deficit contrary to the classic version of the task.

### Aquatic Object Location

To verify this result, we performed an object location task in the swimming pool. In this task (Figures 5B,D), repeated measures ANOVA for the latency to find the hidden platform during acquisition revealed a significant session effect [$\eta_{p}^{2}$ = 0.485; *F*_(3,42)_ = 19.664; *p* < 0.001] but no pre-treatment effect [*F*_(1,14)_ = 0.284; *p* = 0.602], nor session pre-treatment interaction [*F*_(3,42)_ = 0.184; *p* = 0.907]. An ANOVA conducted on the latency to reach the platform during the reactivation trial also did not reveal any pre-treatment effect [*F*_(1,14)_ = 0.457; *p* = 0.510]. These data confirmed that, in general, before treatment all groups of mice learned the position of the platform and displayed the same level of performance during the four learning sessions.

During PT (Figure 5F), all mice cross significantly more time below the object that had remained in a familiar location than the object that had been introduced to a new location. ANOVA on the preference index revealed no treatment effect [*F*_(1,14)_ = 0.242; *p* = 0.630]. Control and propranolol mice exhibited an exploratory preference index that was clearly different from chance level (50%; NaCl: 75.399 ± 9.47%; *p* < 0.001; Propranolol: 81.063 ± 6.50%; *p* < 0.001). Again, propranolol mice presented no memory deficit contrary to the classic version of the object location task.

## Discussion

Results from classic behavioral tasks are summarized in Table 1. In these series of experiments, propranolol treatment induced amnesia if injected during both the consolidation and the reconsolidation phases of the object recognition and object location tasks, only on memory reconsolidation in the passive avoidance and in the CTA tasks and never in MWM regardless of the procedures used.

**Table 1:** **Summary of the behavioral results**.

	Conditioned taste aversion	Passive avoidance	Morris water maze 19°C	Morris water maze 23°C	Object recognition	Object location
Consolidation	No effect	No effect	No effect	No effect	Effect	Effect
Reconsolidation	Effect	Effect	No effect	No effect	Effect	Effect
Aversive task	+++	+++	++	+	0	0
Spatial task	No	No	Yes	Yes	No	Yes
Associative task	Yes	Yes	Yes	Yes	No	No

*For each task, the level of aversiveness (from 0 neutral to +++ very aversive), as well as whether the task is spatial and/or associative is indicated*.

First, we compared within a single study the effect of propranolol on memory consolidation and reconsolidation. In this case, impairing the noradrenergic system with propranolol was more efficient for reconsolidation than for consolidation. Indeed, in aversive tasks, propranolol affected only reconsolidation as it was already described in a passive avoidance task (Przybyslawski et al., 1999) and auditory fear conditioning (Dębiec and LeDoux, 2004). Considering the involvement of the noradrenergic signaling in both consolidation and reconsolidation (Rodrigues et al., 2009), it is surprising that the effect of propranolol appears more robust after reactivation than after the initial acquisition. Moreover, in the two non-aversive object tasks, it was not the case and a clear effect was found both on initial memory consolidation and reconsolidation. One hypothesis advanced by Dębiec and LeDoux (2004) is that “*consolidation of aversive task involves higher intensity of noradrenergic transmission because the negative reinforcement is present and thus requires higher doses of a β-receptor antagonist than other tasks, or other test conditions, such as reconsolidation*”. Thus, the dose of propranolol that effectively blocks memory reconsolidation may not be sufficient to block consolidation (Dębiec and LeDoux, 2004). However, in some cases, propranolol can affect consolidation with a low dose (5 mg/kg) in a mildly stressful task (Cahill et al., 2000). In our experiments, injections were always performed immediately after the end of acquisition or after reactivation. However, the duration of the reactivation was always very short (less than 3 min) but the duration of the training ranged between 15 s for the passive avoidance to 75 min for the MWM. As there are substantial timing effects of the noradrenergic signaling on consolidation (Joëls et al., 2011), maybe the post-training injections of propranolol were too slow to block the fast noradrenergic effects on consolidation whereas post-reactivation injections were still sufficiently quick because of the short reactivation session. Nevertheless, in the present study we found no effect of propranolol injection on memory consolidation in the shortest acquisition task (i.e., the passive avoidance task). Another explanation is that noradrenergic transmission may not be necessary for consolidation, but might be critical for retrieval of aversive task (Murchison et al., 2004). However, some studies reported an amnesic effect of propranolol injection during consolidation in the passive avoidance (Gallagher et al., 1977), in the MWM (Cahill et al., 2000) and in the object recognition (Conversi et al., 2014) tasks. Discrepancies exist in the literature as other studies found exactly the opposite in the same behavioral tasks (Lalumiere et al., 2004; Telegdy et al., 2005; Dornelles et al., 2007; Row and Dohanich, 2008; Okamura et al., 2011; Palotai et al., 2014; and for review, see Roozendaal and McGaugh, 2011).

In all cases, we highlighted the greatest sensibility of the reconsolidation phase to the blockage of the noradrenergic system by the propranolol that strengthens the interest to focus on the reconsolidation process in a therapeutic strategy.

The second objective was to determine the type of memory affected by the propranolol treatment. In the majority of reconsolidation studies, authors have used aversive tasks as passive avoidance or fear conditioning. Using the first task, Przybyslawski et al. (1999) described a clear amnesic effect of propranolol, however, it was not the case in the study by Muravieva and Alberini (2010). Most of fear conditioning studies reported an important memory impairment induced by propranolol injection in both the contextual and the cued fear conditioning (Dębiec and LeDoux, 2004; Abrari et al., 2008; Muravieva and Alberini, 2010) whereas some others did not (Gazarini et al., 2013; Vetere et al., 2013) depending on the protocol or procedures used. The very few studies that have used non-aversive tasks revealed a propranolol-induced amnesia both in the radial maze (Przybyslawski et al., 1999) and in the object recognition task (Liu et al., 2015). In the present work, in comparable experimental conditions in the classic procedures and with the same mouse strain, we obtained a clear amnesia in the most as well as in the least aversive tasks, and not in a moderately aversive task. It is therefore not the level of aversion that influences the effect of propranolol in memory processes.

An alternative explanation is that the effect of propranolol may be linked to the type of the behavioral tasks. The absence of effect of β-adrenergic blocking on the MWM performances may be explained by the spatial nature of this task. However, propranolol affects spatial memory in the object location task. Second, we used associative and non-associative tasks. Again, this factor could not explain the effect of propranolol as in associative tasks as passive avoidance or CTA, we found a great effect, contrasting with the lack of effect in another associative task such as the MWM (see Table 1). The lack of effect of propranolol administered after reactivation may reflect that reconsolidation is not impaired by propranolol, but we cannot exclude that reactivation may fail to induce reconsolidation in the first place. The different results across experiments may reflect different degrees of success in inducing reconsolidation by the various retrieval procedures. Indeed, it has been argued and evidenced some boundary conditions to induce reconsolidation, including a mismatch between memory representation and currently experienced condition, a prediction error or a need for memory updating in human (Sevenster et al., 2012, 2013, 2014) as well as in animal (Lee, 2009; Alfei et al., 2015; Exton-McGuinness et al., 2015). Nevertheless, we observed here an effect of propranolol administration during reactivation on passive avoidance, CTA, as well as in classic object recognition and object location tasks, demonstrating that reconsolidation can be blocked in the present experimental conditions.

Concerning the classic MWM, the reactivation trial consisted in an additional training trial, suggesting no prediction error was generated during memory reactivation, so preventing maybe the induction of reconsolidation. However, in our lab, using exactly the same behavioral procedure used as the present study (Artinian et al., 2007, 2008), we obtained a clear amnesic effect with an injection of protein synthesis inhibitor during reactivation. Thus, in our experimental condition, the reactivation trial induced memory reconsolidation and despite the longer duration of training and the multiple training trials (i.e., a potential difference in memory strength), post reactivation anisomycin impaired subsequent spatial memory performances. However, we cannot completely exclude that propranolol did not affect reconsolidation because the strength of memory and mutiple training acquisition were different in the MWM from another behavioral tasks. With all these data obtained in the same experimental conditions, it is difficult to understand the real role of propranolol in memory consolidation and reconsolidation. None of the factors studied (aversion level, spatial learning, associative learning) seem to explain our behavioral outcomes. However, the MWM is unique in that animals are forced to behave that it is not the case for all other tests. For example, in the object recognition task, maybe the mouse detected the new object, but it was no longer attracted by its novelty. In this case, despite the maintenance of a good contextual memory, behavioral performances will be very poor.

To dissociate between memory problem (i.e., contextual amnesia) and emotional state (i.e., memory valence, detachment…), we developed an aquatic version of the object recognition and the object location tasks. In these two new tasks, the cognitive aspect was exactly the same (i.e., to differentiate between a familiar and a new object or between the locations of two identical objects respectively). However, animals were in water and the only way to escape was to swim to the platform. Thus, in these new procedures, animals were forced to behave.

Unlike classic procedures, propranolol injection during memory reconsolidation had no effect on memory in the object recognition and location tests performed in the aquatic environment. Propranolol-injected mice therefore recognized the familiar object or the familiar location, demonstrating that the ability of dissociation was not affected by propranolol, and so that this treatment did not have a direct effect on the memory reconsolidation of the contextual part of the information.

Our results support data reported in healthy humans showing that propranolol did not affect declarative memory associated to the fear memory but must selectively affect its emotional component (Kindt et al., 2009; Kroes et al., 2010; Schwabe et al., 2013). However, recent data from our group (Lonergan et al., 2013) suggests that an impairing effect can be obtained on both types of memory although the effect of propranolol on the contextual/declarative content seems to be modest in magnitude. PTSD patients, after propranolol treatment, showed a clear reduction of physiologic/emotional responses related to the traumatic memory and almost 70% of patients no longer met the diagnostic criteria for PTSD (Brunet et al., 2011, 2014). In these healed patients, no notable deficit in contextual memory was observed. They still remembered well their traumatic experience, even when the patients were asked to generate from scratch their trauma script at each treatment session (A. Brunet, personal communication, Sept. 14, 2014). Nevertheless, after treatment, there was considerably less distress or negative emotion during the evocation of the traumatic event, as if the patients were now “emotionally detached” from those memories.

Even if we cannot rule out the possible lack of reconsolidation occurence in these experimental conditions, one hypothesis to explain our results in animals is that propranolol may specifically disrupt the reconsolidation of the emotional part of the memory as in human. In this case, no more negative or positive value was associated with the task. Thus, even if propranolol-treated mice remembered the task (intact contextual memory), they would not have been attracted, or on the contrary would have been repelled by the behavioral tests stimuli, unless they were forced to perform, as it was the case in the water environment. In the case of passive avoidance and CTA, the disruption of the emotional memory would have made the dark compartment and the saccharin less aversive for the treated mice. Concerning the object recognition and locations tasks, despite a good discrimination between the two different objects or location, they would not be attracted anymore by their novelty.

However, we have shown this specific effect of propranolol injection on the emotional part of the memory trace only in object tasks, and propranolol may perhaps have an overall effect on memory reconsolidation in other behavioral tests. Moreover, mice in the present study did not undergo any traumatic stress before experiments and thus presented a normal basal noradrenergic level. Given the noradrenergic dysfunction in PTSD (Strawn and Geracioti, 2008), it would be interesting to test the effectiveness of the different protocols of reactivation associated with propranolol on mouse models of PTSD with a noradrenergic hyperactivity. Even so, in human, propranolol seems to affect memory in both healthy subjects (Kindt et al., 2009) and patients suffering from PTSD (Brunet et al., 2011).

## Conclusion

In this sudy, we have shown an action of propranolol administration on the initial consolidation but most importantly on the memory reconsolidation. Moreover, the observed amnesic effect was not related to the aversion level of the task. This effect seems due most likely to a modification of the emotional state of the memory but leave the contextual component of the memory undisturbed. From a treatment perspective and considering the ethical criticisms generated by such an innovative strategy affecting memory (Parens, 2010; Kass, 2003), this represents an ideal state of affairs since most patients do not wish to have their memories “erased” but rather wish they were no longer bothered by them.

## Author Contributions

HV, AB, AD, ML, EM, SP, PR contributed to the acquisition and the analysis of the data. PR contributed to the conception of the work. HV, AB, EB, ABr, PB, PR contributed to the interpretation of the data. All the authors drafted the work or revised it critically, and approved the final version of the manuscript. They all agreed to be accountable for all aspects of the work in ensuring that questions related to the accuracy or integrity of any part of the work are appropriately investigated and resolved.

## Conflict of Interest Statement

The authors declare that the research was conducted in the absence of any commercial or financial relationships that could be construed as a potential conflict of interest.
